# Thyroid hormone-dependent oligodendroglial cell lineage genomic and non-genomic signaling through integrin receptors

**DOI:** 10.3389/fphar.2022.934971

**Published:** 2022-09-05

**Authors:** Rahimeh Emamnejad, Mary Dass, Michael Mahlis, Salome Bozkurt, Sining Ye, Maurice Pagnin, Paschalis Theotokis, Nikolaos Grigoriadis, Steven Petratos

**Affiliations:** ^1^ Department of Neuroscience, Central Clinical School, Monash University, Prahran, VIC, Australia; ^2^ B’, Department of Neurology, Laboratory of Experimental Neurology and Neuroimmunology, AHEPA University Hospital, Thessaloniki, Greece

**Keywords:** integrin, thyroid hormone, oligodendrocyte (OL), Akt, mTOR-mammalian target of rapamycin, monocarboxylate transporter 8 (MCT8)

## Abstract

Multiple sclerosis (MS) is a heterogeneous autoimmune disease whereby the pathological sequelae evolve from oligodendrocytes (OLs) within the central nervous system and are targeted by the immune system, which causes widespread white matter pathology and results in neuronal dysfunction and neurological impairment. The progression of this disease is facilitated by a failure in remyelination following chronic demyelination. One mediator of remyelination is thyroid hormone (TH), whose reliance on monocarboxylate transporter 8 (MCT8) was recently defined. MCT8 facilitates the entry of THs into oligodendrocyte progenitor cell (OPC) and pre-myelinating oligodendrocytes (pre-OLs). Patients with MS may exhibit downregulated MCT8 near inflammatory lesions, which emphasizes an inhibition of TH signaling and subsequent downstream targeted pathways such as phosphoinositide 3-kinase (PI3K)-Akt. However, the role of the closely related mammalian target of rapamycin (mTOR) in pre-OLs during neuroinflammation may also be central to the remyelination process and is governed by various growth promoting signals. Recent research indicates that this may be reliant on TH-dependent signaling through β1-integrins. This review identifies genomic and non-genomic signaling that is regulated through mTOR in TH-responsive pre-OLs and mature OLs in mouse models of MS. This review critiques data that implicates non-genomic Akt and mTOR signaling in response to TH-dependent integrin receptor activation in pre-OLs. We have also examined whether this can drive remyelination in the context of neuroinflammation and associated sequelae. Importantly, we outline how novel therapeutic small molecules are being designed to target integrin receptors on oligodendroglial lineage cells and whether these are viable therapeutic options for future use in clinical trials for MS.

## Introduction

Oligodendrocytes (OLs) are cells that developmentally myelinate axons within the mammalian central nervous system (CNS). The loss of these cells or specific developmental defects in oligodendrogenesis results in denudement of axons, potentiating the brain’s vulnerability to further neurodegeneration (Kotra et al., 2017). Specifically, the integrity of CNS neurons and their axons requires the structural and metabolic support of OLs (Robertson and Moreo, 2016). Support failure results in severe neurological disorders such as that manifested in multiple sclerosis (MS) or during inherited forms of leukoencephalopathy (Feinstein et al., 2015). Indeed, determining how to preserve OLs and their myelin sheaths would be a major contribution to neuroprotection and limit the permanent neurological deficit. To achieve CNS repair through remyelination, one viable option may be therapeutics that modify the diseased tissue environment and can be successfully administered through the tight blood-brain barrier (BBB) and directed to areas of lesions. However, a major limitation is the stalled maturation of endogenous oligodendrocyte progenitor cell (OPC) and the number of cells available to effectively perform repair within viable areas of lesions (Absinta et al., 2019). One main reason for remyelination failure in MS is stalled OL differentiation that is partially due to a lack of trophic support (Australia, 2022).

It is now evident that thyroid hormones (THs) are transported across the plasma membrane by specific TH transporters such as monocarboxylate transporter 8 (MCT8, encoded by the *SLC16A2* gene) (Friesema et al., 2003). MCT8 was first identified in the brain and is highly expressed by neuronal populations of the cerebral and cerebellar cortex, hippocampus, striatum, and hypothalamus (Heuer et al., 2005); however, functional studies performed in *Xenopus laevis* oocytes elucidated its importance in TH transport (Friesema et al., 2003). In fact, MCT8 plays a critical role in brain development, particularly in the human brain, where it facilitates the uptake of T3 across the blood–brain barrier (BBB) (Ceballos et al., 2009). The monocarboxylate transporter 10 (MCT10), encoded by the *SLC16A10* gene, has also been shown to transport TH in addition to performing its common T-type amino acid transporter function (Friesema et al., 2008). MCT10 shows overlapping expression with MCT8 and has recently been identified as an important transporter expressed within developed white matter tracts of the mouse brain (Friesema et al., 2008), which suggests a functional role in mature OLs. TH plays a central role in OL development and myelination *in vivo* by regulating the expression of specific maturation genes, such as myelin basic protein (MBP), and thereby regulates myelination (Younes-Rapozo et al., 2006). Furthermore, T3 affects the critical replication of OPCs and the myelin production and survival of OLs, which are developmentally-regulated events that lead to coordinated myelination (Fernandez et al., 2004).

## Treatment options and therapeutic limitations

Within the past 29 years of disease-modifying therapies (DMTs) development and since the introduction of interferon-β (IFN-β) as a treatment for relapsing remitting MS (RRMS) (1993), there have been few therapeutic options for both primary progressive MS (PPMS) and secondary progressive MS (SPMS) (Feinstein et al., 2015; Robertson and Moreo, 2016; Kotra et al., 2017). Currently, 16 Therapeutic Goods Administration (TGA) and food and drug administration (FDA)-approved therapies exist with the intention of reducing clinical and sub-clinical disease activity, which otherwise contributes to long-term disability (Absinta et al., 2019; Australia, 2022). Additionally, existing therapies targeting inflammation in RRMS have proven effective in managing relapse rates. Generally, protection against relapse is 20–35%, 50–55%, and >60% in injectable, oral, and infusion therapies, respectively (Hauser et al., 2013). However, abrogating neurodegenerative processes in PPMS and SPMS, independent of inflammatory lesion activity (Ontaneda et al., 2017), remains ineffective. Examples are chronic active plaques seen in progressive disease, which persist unabated, and are associated with an aggressive disease-course and poor clinical outcomes (Absinta et al., 2019).

Despite the emergence of radiological indicators, the sole aim of DMTs is to manage symptomology and inflammation, but a lack of efficacy in treating progressive forms of MS indicates that progressive disease treatments remain an unmet medical need. Furthermore, none of the current therapies provide neuroprotection or neurorepair, which is required to prevent future neurological deficits (Robertson and Moreo, 2016). Consequently, recovery from progressive myelin degeneration remains unattainable (Sen et al., 2022).

However, one plausible avenue to attain neuroprotection and repair is to promote thyroid hormone (TH) signaling in OLs.

## Thyroid hormone synthesis and regulation

TH are endocrine messengers that regulate several physiological processes, such as development, growth, and metabolic activities, in all mammalian cells (Brent, 2012). In vertebrates, particularly humans, TH is crucial for effective brain development and functioning by promoting the maturation of neuronal and glial cells while maintaining their cytoarchitecture (Salazar et al., 2019). Additionally, it stimulates the proliferation, differentiation, and migration of neural cells that target the formation of synaptogenesis, dendritic cell branching, and myelination (Bernal et al., 2000). Therefore, TH is a chief regulatory hormone in the homeostatic functioning of the developing and adult brain.

The physical structure of TH includes two main forms as follows: the pro-hormone as 3, 5, 3′, 5′-L-tetraiodothyronine or thyroxine (T4) and its active form with one less iodine atom as 3, 5, 3′-L-triiodothyronine (T3) (Bernal, 2005). The conversion of T4 to T3 can be achieved by selenocysteine-dependent deiodinase (DIO) enzymes that catalyze the removal of iodine (deiodination) and activate T4 to T3 via DIO2. Importantly, more than 80% of cellular T3 is derived from the deiodination from T4 by tissue-specific DIOs (Larsen et al., 1981; Pilo et al., 1990; Maia et al., 2005). The expression of DIO2 is restricted to the CNS (Visser et al., 1982), pituitary (Maeda and Ingbar, 1984), brown adipose tissue (Leonard et al., 1983), and placenta (Kaplan and Shaw, 1984). In the rat and human brain, there is differential expression of DIO2 in the hippocampus, cortex, and corpus callosum (Croteau et al., 1996), and at the cellular level, DIO2 is mostly expressed in tanycytes, astrocytes, OLs, and interneurons (Guadaño-Ferraz et al., 1997; Tu et al., 1997; Guadaño-Ferraz et al., 1999). This suggests that these supporting glia are the first port of call for converting thyroid hormone to the active form for intracellular metabolism that ensues within the axons and neurons they support. Alternatively, where DIO2 activates T4 to T3, the inactivation of T3 to rT3 can occur via specific DIO3 activity (Schweizer et al., 2014) that is also located within astrocytic and OL cells of the CNS (Bernal, 2007). This demonstrates the importance of these macroglia in governing the homeostatic regulation of TH within the brain.

The synthesis and secretion of TH are both regulated by the hypothalamic-pituitary-thyroid (HPT) axis that utilizes a negative feedback loop system (Medici et al., 2015) to maintain a homeostatic environment of T4 and T3 (Mendoza and Hollenberg, 2017). Environmental and physiological stimuli trigger the synthesis of TH, whereby thyrotropin-releasing hormone (TRH) neurons in the paraventricular nucleus (PVN) of the hypothalamus secrete TRH in the median eminence (Kopp, 2001; Medici et al., 2015). As a result, TRH binds to the TRH receptor located on the membrane of thyrotrophs to release thyroid-stimulating hormone (TSH) from the anterior-pituitary (Kopp, 2001; Medici et al., 2015; Mendoza and Hollenberg, 2017). TSH then acts through its specific receptor (the TSH receptor) to stimulate the growth and function of thyroid follicular cells, which regulate the synthesis and secretion of T4 and T3 from the thyroid gland and into the circulation (Kopp, 2001), with T4 constituting approximately 80% of that secreted into the circulation (Pirahanchi et al., 2021). To ensure efficient systemic TH delivery, the majority of TH are bound to specific plasma proteins (albumin, thyroxine-binding globulin, and transthyretin) of varying binding affinities and dissociation rates to create redundancies in the TH transport network (Schreiber and Richardson, 1997).

## Thyroid hormone signaling drives oligodendrocyte differentiation and myelination

OLs are mature glial cells responsible for ensheathment of axons with myelin in the brain and permit rapid saltatory conduction between neurons (McTigue and Tripathi, 2008; Kim and Petratos, 2019). OPC are derived from neural precursor cells (NPCs), namely, glial fibrillary acidic protein (GFAP)-positive astrocytes (type-B cells) that originate in the subventricular zone (SVZ) adjacent to the lateral ventricle in the brain (Menn et al., 2006). These then migrate to the corpus callosum (cc), striatum, and fimbria fornix via platelet-derived growth factor (PDGF) signaling that promotes indiscrete motility of OPCs; furthermore, there is interaction with the basement membrane molecules, such as laminin (LN), in the extracellular matrix (ECM) (Tsai et al., 2016; Suzuki et al., 2019).

**FIGURE 1:**
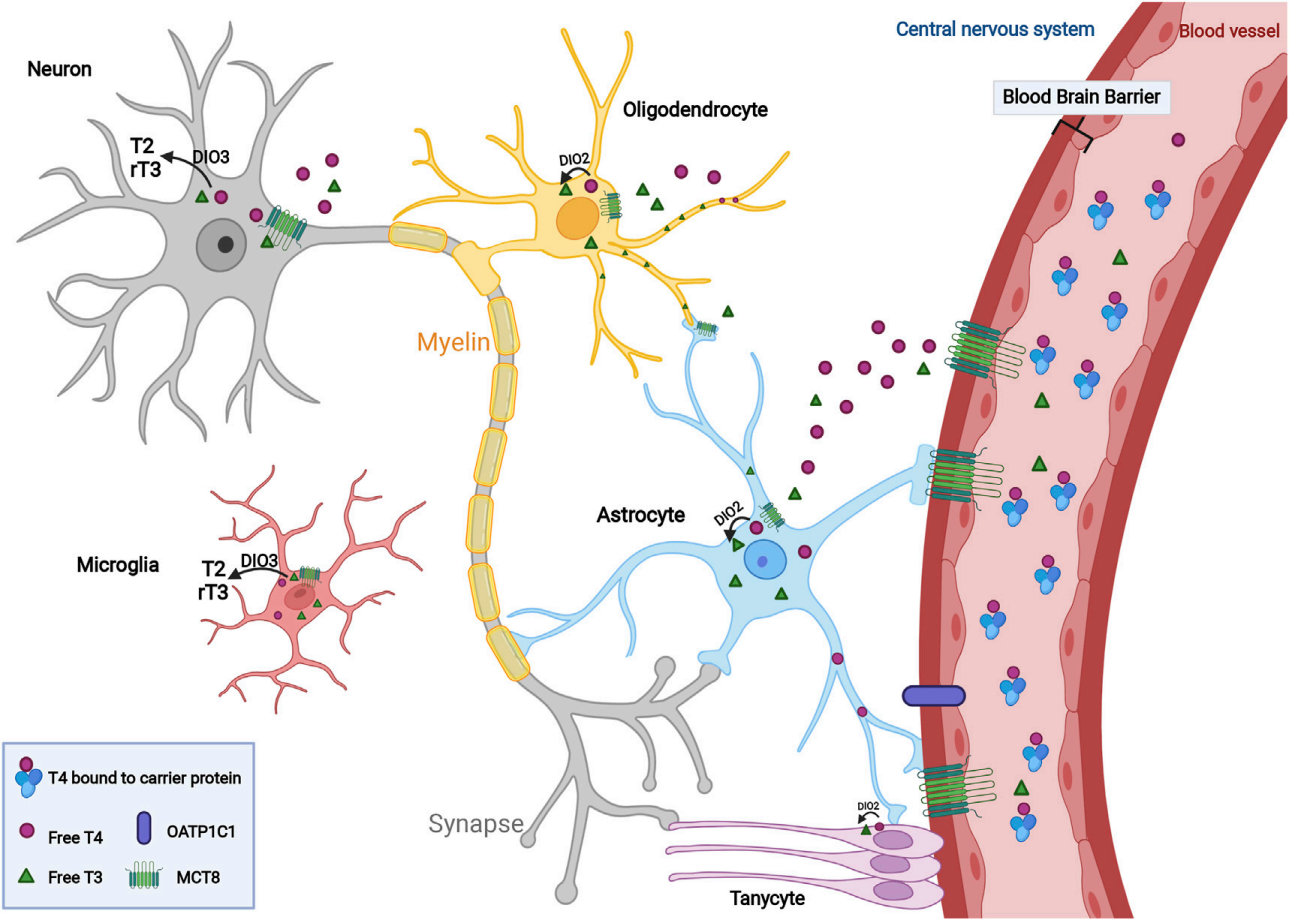
Transport and regulation of thyroid hormone within the central nervous system. In the circulation, a higher ratio of TH is in the form of free T4 compared to T3 level, which is sustained in an inactive status due to its binding with carrier protein such as albumin. T4 and T3 cross the blood-brain barrier (BBB) via the monocarboxylate transporter 8 (MCT8) and OATP1C1 and then transported into neurons and glial cells including astrocytes, oligodendrocytes, microglia and tanycytes within the CNS. To promote cellular proliferation, differentiation and myelination mediated by TH, T4 converts to its active form, T3, in the presence of deiodinase type 2 (DIO2). Active T3 mediates myelination by mature oligodendrocytes. Increased thyroid hormone concentrations contribute to the conversion of T3 and T4 to inactive metabolites, T2 and rT3, to maintain endogenous homeostasis under the regulation of DIO3.

OPCs proliferate in response to both PDGF and fibroblast growth factor (FGF) with downregulation of the PDGF-α receptor that initiates differentiation to late progenitor cells (Baron et al., 2000). This eventually results in non-myelinating and myelinating OLs that are implicated in the repair of myelinated tracks (Petratos et al., 2004). These mature OLs extend and spirally wrap their lipid-rich membrane around neuronal axonal segments several times to form the myelin sheath and promote lower energy consumption and faster saltatory impulse conduction during neuronal communication (Pfeiffer et al., 1993; Zalc and Colman, 2000; Hartline and Colman, 2007; Nave, 2010; Lebel and Beaulieu, 2011; Hughes and Appel, 2016). As a result, the CNS quickly and efficiently acts to maintain the functional integrity, metabolism, and long-term survival of neurons and improves motor-sensory function, cognition, and neuroplasticity (Nave, 2010; Lebel and Beaulieu, 2011; Saab and Nave, 2017) (Figure 1).

Following the occurrence of a demyelinating lesion in the cc, SVZ-derived NPC populations increase in number, as reflected in cuprizone models of demyelination, with increased NPCs that contribute to neuroprotection (Fancy et al., 2004; Nait-Oumesmar et al., 2007; Nait-Oumesmar et al., 2008; Butti et al., 2019). This highlights OL maturation in progressive disease as an intuitive target with the capability of leveraging the already present population of OLs in the cc and inducing differentiation to mature myelinating OLs that permit remyelinating events to occur.

Remyelination can be induced through TH signaling, which acts as a regulatory trophic signal of OL maturation and subsequent myelination through terminal differentiation of OPCs into myelinating mature OLs; this further occurs through rapid cell-cycle arrest that is elicited through genomic and non-genomic signaling (Lee and Petratos, 2016; Kim and Petratos, 2019).

## Thyroid hormone-dependent genomic signaling

Genomic TH signaling via TH receptors (TRs), TRα, and TRβ, which are expressed in the nucleus of OLs, are integral in the gene transcription involved in differentiation and following TH binding (Billon et al., 2001; Lee and Petratos, 2016). Furthermore, cytoplasmic T3 binds to TRα, TRβ1, and TRβ2. These TRs form heterodimers with other TRs such as the retinoid X receptor (RXR). Increased levels of TR-RXR heterodimerization located in oligodendroglia (Pombo et al., 1999) can actively increase binding to TH response elements (regulatory DNA sequences) (Cheng et al., 2010; Taylor and Heyland, 2017) and drive gene transcription, i.e., TH-dependent transcriptional activity (Hsu et al., 1995).

TRα is implicated in the initial cell-cycle exit and is predominantly expressed in OPCs, whereas TRβ1 occurs in later-developmental stages (pre-OLs) and is involved in terminal differentiation and myelination (Baas et al., 1998; Billon et al., 2002; Bury et al., 2002; Wallis et al., 2010; Lee and Petratos, 2016). Furthermore, the absence of TRα and TRβ results in persistent proliferation of OPCs and indicates their synergistic role in normal OL development (Baas et al., 2002). Despite the functional conclusions made through *in vivo* studies of TRβ, its role *in vivo* still remains largely unknown (Dong et al., 2009).

There is further confounding of genomic signaling where un-liganded TRs may repress T3 responsive genes. This is particularly important in the context of SVZ-derived OPCs where unbound TRs in hypothyroid conditions recruit the nuclear co-repressor responsible for repression of genes involved in OL differentiation from NSCs observed during development. Such repression can be associated with upregulation of histone deacetylases (HDACs), which repress myelin gene expression through increased inaccessibility of DNA to transcription factors via removal of acetyl groups from histones (Castelo-Branco et al., 2014a; Wheeler et al., 2015). However, the maintenance of neural cells involved in embryonic development are also regulated by T3 in adults. This was shown in adult hypothyroid rodents and increased proliferation of SVZ cells (Lemkine et al., 2005; Lopez-Juarez et al., 2012).

## Thyroid hormone signaling in MCT8-deficient circumstances

Thyroid hormone transporters (THTs) that are localized to plasma membranes include MCT8 (*SLC16A2*), a high affinity TH-specific transporter of both T3 and its pro-hormone T4, that facilitates the passage of these insoluble hormones across the BBB with great efficacy into the CNS to exert neurobiological effects (Friesema et al., 2003; Ceballos et al., 2009; Kinne et al., 2010; Vatine et al., 2017). In addition, the homolog, MCT10 (49% amino acid overlap), is also capable of T3 uptake and efflux (Friesema et al., 2003; Friesema et al., 2008). These transporters exhibit the highest substrate specificity to TH (Bernal, 2006) relative to other known alternative THTs, such as L-type amino acid transporters (LATs) (Mayerl et al., 2014) and the organic anion transporter (OAT) family (Sun et al., 2014), present in other species.

Genomically, it has been identified that THs rely on THTs, such as MCT8, to elicit TH-dependent differentiation in OLs (Vatine et al., 2021). The MCT8s function is elucidated not only in T transport at the BBB, but locally at OL plasma membranes (Lee and Petratos, 2016; Lee et al., 2017).

In instances of *SLC16A2* mutation, the dysfunction of MCT8 results in insufficient intracellular levels of T3 in the presence of increased serum free levels, which results in hypomyelination and the severe psychomotor retardation seen in Allan-Herndon-Dudley syndrome (AHDS) (Friesema et al., 2004; Armour et al., 2015; Valcárcel-Hernández et al., 2022). MCT8 dysfunction, as a prerequisite of AHDS, is supported by limited pre-clinical data from mouse models and clinical trials in AHDS patients and when treated with the TH analog 3,5-diiodothyropropionic acid (DITPA) resulted in amelioration of peripheral hyperthyroidism and overall hypermetabolism (Verge et al., 2012; Ferrara et al., 2015).

The TH treatment in animal models of MS yields similar beneficial effects and permits a reduction in demyelination and axonal injury through anti-inflammatory properties (Calza et al., 2002; Dell'Acqua et al., 2012). In addition to its ability to accelerate remyelination following acute cuprizone administration (Zendedel et al., 2016; Hartley et al., 2019), there is emerging evidence from our group suggesting that MCT8 is also downregulated in neuroinflammatory conditions seen in MS. This is similar to those observed in animal models of MS where the acute inflammatory-mediated downregulation of MCT8/10 is observed throughout the CNS of SPMS Chronic-active Lesion (preliminary data). Therefore, genomic signaling in a TH-dependent manner via MCT8 is not a viable treatment option.

However, the thyroid hormone analog, DITPA has been shown to function in an MCT8-independent manner as suggested earlier in treating patients clinically identified as having hyperthyroidism as a consequence of AHDS (Verge et al., 2012). Indeed, in the *slc16a2* ^−/−^ zebrafish model of AHDS, the administration of DITPA also led to the restoration of myelin and normalization of locomotor behavior (Zada et al., 2014). These paradigms are consistent with our own findings in a cell culture model that utilized human embryonic stem cell hESC-OPCs. In this case, the administration of the small molecule DITPA [AU2015372427B2, 2020-07-02 (Granted), EP3237605B1, 2021-02-24 (Granted), US10640748B2, 2020-05-05] (Granted) led to OPC differentiation, maturation, and subsequent myelination. In addition, salvaging TH-deficient OPCs permits their maturation to myelinating OLs (Lee et al., 2017).

Therefore, it is postulated that OL-specific MCT8 deficiency can uncouple TH-dependent genomic signaling and thus the lack of differentiation of myelin-producing OPCs that may ensue promote the development of neurological diseases such as MS (Kim and Petratos, 2019). This hypothesis is currently under investigation within our laboratory.

## The role of integrins as alternate thyroid hormone-dependent receptors

The function of the ECM involves a complex interplay between several extracellular molecules that collectively govern the structural integrity of tissues and organs while providing a selective and protective barrier between the extracellular and intracellular environment (Pompili et al., 2021). Additionally, the ECM is essential for regulating cellular processes such as growth, migration, differentiation, and adhesion (Plow et al., 2000; Hynes, 2009; Barczyk et al., 2010; Wen et al., 2019; Pompili et al., 2021). The composition of the ECM encompasses glycosaminoglycans (GAGs), proteoglycans, growth factors, and other ECM proteins, such as collagen and adhesion proteins, where the latter includes Laminin LN that binds to receptors on the ECM that are known as integrins (Bonnans et al., 2014; Wen et al., 2019).

Integrins are transmembrane heterodimers consisting of *α* and *β* subunits linked by a non-covalent bond that modulates the interaction between the ECM and surrounding cells (Campbell and Humphries, 2011). In mammals, there are 24 different integrin receptors formed from a combination of 18*α* and 8*β* subunits (Hynes, 2002), which are displayed as two membrane-spanning helices (one for each subunit) and a small cytoplasmic tail located on the basement membrane of the ECM (Ruppert et al., 1995; Montgomery et al., 1996; Campbell and Humphries, 2011).

The biological function of integrin receptors acts as an adhesion molecule and contributes to the action of the ECM by providing cell-cell, cell-ECM, and ligand binding (Plow et al., 2000; Campbell and Humphries, 2011; Wen et al., 2019; Pompili et al., 2021). These structural and pleiotropic interactions include cell surface adhesion proteins, soluble vascular cell adhesion molecule , and ECM ligands such as vitronectin, fibronectin, vascular cell adhesion molecule VCAM-1, and mucosal addressin cell adhesion molecule MAdCAM-1 (Plow et al., 2000; Campbell and Humphries, 2011; Wen et al., 2019; Pompili et al., 2021). Thus, integrin receptors contribute to the structural maintenance and integrity of tissue, cellular trafficking, differentiation, migration, and adhesion (Plow et al., 2000; Barczyk et al., 2010). Furthermore, integrins play a critical role in the immune system by enabling leukocyte trafficking, with the migration and formation of the immunological synapse, and phagocytosis during adaptive and innate immune responses (Rocha-Perugini et al., 2016).

OLs display a high expression of several integrins with distinct functions. These include αvβ8 integrin expressed during oligodendroglial development, while αvβ1, α6β1, αvβ3, and αvβ5 integrins are sequentially expressed during OL differentiation *in vitro* (Milner and Ffrench-Constant, 1994). For example, during differentiation, the expression of αvβ3 integrin is strictly regulated, where at its highest peak, it can enable the transition from O2-A progenitors to post-mitotic OLs (Milner and Ffrench-Constant, 1994; Lee and Petratos, 2016). Comparatively, β1 integrin is also heavily involved during OL development. One study utilized a ligand-integrin modified alginate hydrogel in a three-dimensional culture to demonstrate the proliferation and differentiation of NPCs into OLs (Wen et al., 2019). In this study, it was determined that the interaction of ligand LXY30 with an α3β1 integrin receptor promoted human neural precursor cells hNPCs toward OL lineage cells, whereas the ligand LXW64 interaction with the αvβ3 integrin receptor shared a similar but less potent differentiation outcome (Wen et al., 2019). Furthermore, the conditional ablation of the β1 integrin gene in OPCs demonstrated OL survival but not axon ensheathment, myelination, or remyelination as seen in the lysolecithin model of demyelination (Benninger et al., 2006). Another study that utilized the dominant-negative inhibition of β1 integrin in OPCs identified α6β1 integrin as a driver for myelination (Relvas et al., 2001). Moreover, its interaction with LN promoted *in vitro* and *in vivo* OL survival (Frost et al., 1999; Colognato et al., 2002).

In the human genome, LN is composed of *α*, *β,* and *γ* subunits where the *α* subunit is encoded by 5 genes and *β* and *γ* subunits by 3 genes (Aumailley, 2013). A combination of these chains generated a total of 16 heterotrimeric shapes of LN (Aumailley, 2013).

Similar to the oligodendroglial lineage in the CNS, Schwann cell differentiation and myelination in the peripheral nervous system (PNS) is also mediated by interactions between LN and β1-integrin as supported by the conditional ablation of β1 integrin (Feltri et al., 2002). However, the major difference is the availability of LN. For example, in the PNS, it is readily sourced from the basal lamina for Schwann cells, while in the CNS, it only becomes available for α6β1 integrin just prior to myelination (Colognato et al., 2002). Whether this is universally observed for all other integrins in the CNS is yet to be determined.

## Role of thyroid hormone on oligodendroglial development and myelination via integrin-mediated signaling

Given the importance of integrins in regulating oligodendroglial development, a major ligand that drives this action is TH (Baas et al., 1997). Therefore, the precise level and timing of TH stimulation can modulate cell-specific stages of development, proliferation, differentiation, myelin production, and survival of oligodendroglia (Jones et al., 2003; Younes-Rapozo et al., 2006; Williams, 2008). This premise is supported by a study confirming that the serum level of TH is at its peak during the active myelination phase of the fetal and postnatal stages demonstrating that TH controls the timing and development of OLs (Sarlieve et al., 2004).

Under hypoxic-ischemic (HI) injury conditions, treatment with T4 was shown to significantly alleviate OL death and hypomyelination (Hung et al., 2018). Moreover, its role in hypoxia-related white matter pathologies involved the stimulation of oligodendrogenesis and the regulation of OL metabolism by inducing angiogenesis, which indicates its importance in maintaining OL viability (Yuen et al., 2014). Data has also implicated T4 as a modulator of WM injury from the premature brain by promoting OPC proliferation during early developmental stages, followed by changes to the morphology of post-mitotic OLs, which is a maturation process that increases myelination (Hung et al., 2018).

Given the action of T4, the effect of T3 (the active form of TH) has also been observed *in vivo* and *in vitro*. A comprehensive study in rats demonstrated that OPCs, which were observed under conditions of acute and chronic T3 deficiencies, produced higher numbers of pre-OLs but lower numbers of mature OLs (Younes-Rapozo et al., 2006). Similarly, an *in vitro* study on the cerebral cortices of rat neonates with T3-deficiency revealed a deprivation of the cytoplasm-filled myelin membrane. This finding suggests the importance of T3 during the later stages of OL development in the maturation of myelination (Younes-Rapozo et al., 2006), where the latter is supported by the upregulation of myelin gene expression, including myelin basic protein (MBP) and proteolipid protein (PLP), during T3 administration (Younes-Rapozo et al., 2006).

Collectively, these data propose that T4 and T3 act as mitogens and can induce development, differentiation, and maturation while regulating the cell cycle of OLs that result in the formation of the complex myelin membrane around neuronal axons (Kim and Petratos, 2019). However, their bioavailability is controlled by TH transporters, DIOs, and TH receptors in PNS and CNS tissues (Mendoza and Hollenberg, 2017).

## Potentiation of Akt/mTOR signaling by the synthetic thyroid hormone analog

Given that DITPA signals independently of MCT8, its mechanism of action remains largely unknown. However, it has been postulated that the effects of DITPA are due to activation of the non-genomic pathways, Akt and MAPK (mitogen-activated protein kinase), through integrin αvβ3, which is a known non-genomic receptor (Lee et al., 2017). TH can act via the αvβ3 integrin receptor and upregulate activity in the Akt/mTOR signaling pathway (Cao et al., 2005; Cheng et al., 2010). However, one major criticism of those studies positing the non-genomic actions of DITPA is the lack of consideration of this pathway. Moreover, despite MCT8 ablation, no consideration was made in confirming the ability of DITPA to bind to integrin receptors through use of the integrin-antagonist arginylglycylaspartic acid (RGD peptide) (Plow et al., 2000; Davis et al., 2021).

**FIGURE 2:**
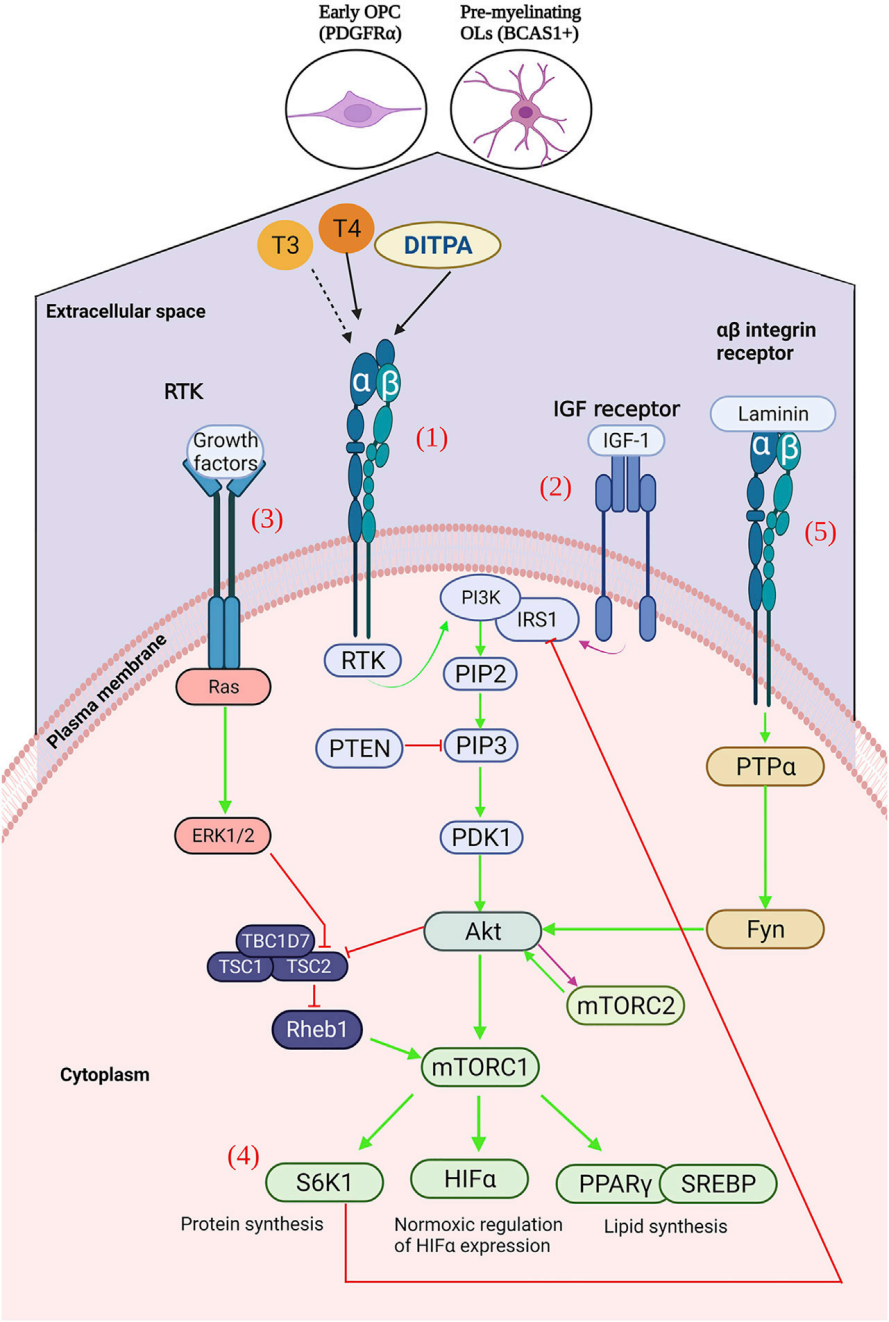
(1) Ligation of T3, T4 or DITPA to αβ integrins results in the activation of receptor tyrosine kinase (RTK), activating an intracellular signalling cascade – activating 3-phosphoinositide 3 kinase (PI3K), of which leads to the generation of Ptdlns(4,5)P3 (PIP3). PIP3 is then able to activate phosphoinositide-dependent kinase-1 (PDK1), which results in Akt activation, and subsequent mTOR upregulation128. Downstream activation of S6K1 (protein synthesis), HIFα (glycolytic metabolism), PPARγ and SREBP (lipid synthesis). (2) mTORC2 is mainly activated by growth factors, such as IGF-1, which can directly activate PI3K via the insulin-receptor substrate-1 (IRS-1) – leading to the downstream activation of mTORC2130. (3) Activation of the receptor tyrosine kinase by growth factors, leads to the activation of the Ras signalling cascade, and subsequent Erk1/2 activation – resulting in TSC2 inhibition and consequently mTORC1 upregulation. (4) S6K1 inhibits IRS-1, leading to subsequent ERK2 inhibition and consequent downregulation of S6K1. (5) Laminin binding to αβ integrins results in (Created with BioRender.com).

This is vital because two binding sites exist on the integrin αvβ3 for TH (Figure 2). The S1 site binds T3 resulting in PI3K activation; the S2 site predominantly binds T4 and T3 to a lesser extent and is involved in activation of MAPK (Davis et al., 2021). Hence, using the RGD peptide permits delineation between these non-genomic signaling pathways. However, an increase in Akt phosphorylation was seen following DITPA administration to hESC-derived OPCs and in the event integrin αvβ3 could be implicated to be a result of S1 binding (Lee et al., 2017).

## Non-genomic initiation of the Akt/mTOR signaling pathway

Non-genomic TH-dependent signaling, independent of TR-T3 complexes, involves binding of T4 to integrin αvβ3, which is a plasma membrane receptor present in most vertebrate cells (Taylor and Heyland, 2017). This results in translocation of the TRβ1 receptor from the cytoplasm to the nucleus and permits signal transduction through the tyrosine kinase/PI3K/Akt/mTOR pathway (Lin et al., 2005; Moeller et al., 2006; Moeller et al., 2011) (Figure 2). The mTOR protein is a serine/threonine kinase and a central coordinator of cell anabolism and catabolism. It is comprised of two distinct protein complexes as follows: mTOR complex 1 (mTORC1) and mTOR complex 2 (mTORC2) (Saxton and Sabatini, 2017). Complex mTORC1, which possesses a higher sensitivity to its antagonist rapamycin, contains adaptor protein raptor (regulatory associated protein of mTOR), and mTORC2 contains the adaptor protein Rictor (rapamycin-insensitive companion of mTOR), which showcases greater tolerance but similar inhibition at protracted high doses (Sarbassov et al., 2006; Lamming et al., 2012). mTORC1 predominates during cellular growth and is activated through phosphorylation of downstream effector molecules involved in mitochondrial, glycolytic, and lipid metabolism. In mTORC2, it is implicated in the cytoskeletal organization of cells and is a contributor to the complete activation of Akt (Sarbassov et al., 2005; Guertin et al., 2006).

Akt is a serine/threonine protein kinase responsible for the phosphorylation and regulation of substrates involved in principal cellular functions (Manning and Cantley, 2007). T4 signaling downstream of integrin αVβ3 binding results in activation of mTOR via the Akt/PI3K/mTOR pathway. Akt can activate mTORC1 directly or through inhibition of TSC2 (tuberous sclerosis complex 2), which is a guanosine triphosphate hydrolase (GTPase)-activating protein that is responsible for the inhibition of the Ras family GTPase (Rhes). Otherwise, it is responsible for the activation of mTORC1 following Akt signaling (Zou et al., 2011). The extracellular signal-regulated kinase 1 (ERK1)/MAPK pathway is another notable signal transduction mechanism shown to inhibit TSC2 and results in mTOR activation (Lin et al., 2009; Gaesser and Fyffe-Maricich, 2016).

Furthermore, LN acting via αβ integrins results in protein tyrosine phosphatase *α* (PTPα)-Fyn activation and subsequent Akt activation (Ye et al., 2008). In addition to insulin growth factor-1 (IGF-1) acting at the IGF-1 receptor (IGF-1R), it can also activate the Akt signaling pathway (Manning and Cantley, 2007). However, mTORC2 can be regulated through growth factor-dependent signaling that may include IGF-1 (Yuan et al., 2012) (Figure 2). It is postulated that the thyroid hormone analog DITPA can act in a TH-dependent manner to promulgate OL differentiation, proliferation, maturation, and remyelination via the Akt/mTOR signaling pathway (Gaesser and Fyffe-Maricich, 2016).

## Akt in differentiation and myelination

Several studies have demonstrated the activation of Akt and its involvement in myelination. This was demonstrated in experiments by Goebbels et al. (2010) whereby the PI3K/Akt inhibitor, phosphatase, and tensin homolog (PTEN) were conditionally knocked out in transgenic mice, which resulted in hypermyelination of the CNS concordant with increased levels of Akt phosphorylation. However, no increase was observed in mature OLs (Goebbels et al., 2010). This suggests that Akt activation is associated with CNS myelination but not remyelination associated with the actions of mature myelinating OLs. However, given that the OPC population was not tested, the involvement of Akt in differentiation during early brain developmental stages cannot be ruled out. Furthermore, a study by Chan et al. (2014) established that increased Akt activity was associated with an increased population of OPCs, which could later serve as myelinating OLs in remyelination (Chan et al., 2014).

Affirmation of the effects of Akt signaling on myelination was procured through combinative administration of PTEN and growth factor administration, which is a known activator of Akt and resulted in MAPK activation, in addition to mTOR downstream, and confirmed the involvement of Akt and parallel activation to MAPK in myelination and mTOR as a downstream effector (De Paula et al., 2014). The proposed regulatory role that mTOR has on myelination was further supported following a reduction in hypermyelination after rapamycin treatment in constitutively active Akt mice (Narayanan et al., 2009).

## mTOR in differentiation and myelination

mTOR is the primary effector mechanism in Akt-induced myelination (Narayanan et al., 2009) and permits enhanced myelination and myelin protein expression in accordance with upregulated mTORC1 activity (Flores et al., 2008). The inactivation of mTOR leads to decreased myelin RNA and protein levels (Bercury et al., 2014). Furthermore, mTORC1 may be implicated to a greater degree in MBP expression and the mTORC2 influence on myelin gene expression (Tyler et al., 2009). This suggests the function that mTOR has in transcription and translation during OL development.

Validation of the maturation role performed by mTOR was derived from three studies performing conditional ablation of mTORC1 in 2′, 3′-cyclic-nucleotide 3′-phosphodiesterase (CNP)-Cre; Raptor, Olig1-Cre; Rheb1, and CNP-Cre; Rheb1 mice that resulted in hypomyelination (Bercury et al., 2014; Lebrun-Julien et al., 2014; Zou et al., 2014). In addition, OPC differentiation defects occurred in two studies (Bercury et al., 2014; Zou et al., 2014) but not the third (Lebrun-Julien et al., 2014). The important role of mTORC1 in myelin maintenance with respect to the dynamic nature of myelin in adult CNS was shown by Lebrun-Julien et al. (2014), whereby mTORC1 ablation resulted in hypomyelination but mTORC2 did not (Gaesser and Fyffe-Maricich, 2016).

While mTORC1 may modulate myelination, there is disagreement concerning the role of mTORC2 in both myelination and OL differentiation. It was demonstrated by Bercury et al. (2014) that the absence of myelin delay at P14 (myelinating OLs) was insignificant compared to mTORC1 following its ablation; however, OL development was accelerated–with a decrease in OPCs and an increase in mature OLs (Gaesser and Fyffe-Maricich, 2016). Conversely, Lebrun-Julien et al. (2014) demonstrated hypomyelination at P14 but no alteration in OPC differentiation. This may be due to the role of mTORC2 in myelin gene expression opposed to MBP expression, or was perhaps due to a greater implication of mTORC2 in the regulation of OL process dynamics through cytoskeletal reorganization and MBP localization (Sarbassov et al., 2005; Guertin et al., 2006; Musah et al., 2020).

## Interplay between Akt/mTOR and MAPK signaling

Despite the intricacies of each mTOR complex, it is posited that both mTORC1 and mTORC2 have distinct roles in OL differentiation, specifically the transition of late progenitor cells to immature OLs (Tyler et al., 2009). This follows the synergistic relationship between Akt/mTOR and ERK1/2 signaling, whereby two points of convergence exist between these pathways determining OL differentiation, namely, at the level of the insulin receptor substrate-1 (IRS-1) and in addition to S6K1 (p70S6K) (Gaesser and Fyffe-Maricich, 2016). Whereby S6K1 inhibits IRS-1 and prevents ERK1/EKR2 activation, ERK2 inhibition results in S6K1 downregulation (Gaesser and Fyffe-Maricich, 2016). Thus, for proper differentiation to occur from OPCs to pre-myelinating OLs (ERK1/2 driven) and pre-myelinating to mature myelinating OLs (Akt/mTOR driven), some homeostasis must exist between the two signaling pathways (Gaesser and Fyffe-Maricich, 2016).

## Akt/mTOR signaling in remyelination

There are limited studies demonstrating the ability of Akt signaling in remyelinating events in the CNS or in OL cultures. However, recent significant evidence indicates that the Akt/mTOR pathway may regulate remyelination following spinal cord injury and convert astrocytes to OL lineage cells (Ding et al., 2021). However, the role of the Akt/mTOR pathway remains disputed by a few studies and suggests that its activation may confer a negative immunomodulatory outcome with no therapeutic potential in its ability to induce remyelination (Mammana et al., 2018).

In the circumstance that Akt/mTOR signaling does induce myelination and differentiation of OPCs, two principal limitations associated with the potentiality of remyelinating events in MS can be addressed. The first was the stalled maturation of endogenous OPCs; the second was an insufficient number of OPCs to successfully conduct neurorepair (Sher et al., 2008).

## Possible pre-conditions of integrin signaling by mTOR

Integrin receptors are dormant up until their activation with the mechanism of TH-induced activation still widely unknown. One plausible pre-condition of integrin activation in the context of mTOR signaling is the hypoxic induction of integrin receptors (Davis et al., 2021). Hypoxia-inducible factor-1 alpha (HIF-1α) activates transcription of genes facilitating integrin avβ3 (Sesé et al., 2017). Furthermore, Akt/mTOR signaling acts as a condition of normoxia to stabilize hypoxic HIF-1α expression (Corcoran and O’Neill, 2016; Davis et al., 2021). A hypoxic microenvironment may be favorable for remyelination, as the chronic inflammatory milieu (seen in early stages of MS) has been shown to promote the removal of HIF-1α^+^ monocytes in the hypoxic microenvironment and thereby facilitate neurorepair (Kotter et al., 2001; Bieber et al., 2003). In a rodent model where suppression of acute inflammation via corticosteroid administration led to delayed remyelination, no effect on OPC migration toward lesions was observed and the inhibitory effects exerted upon oligodendroglial cell differentiation is argued to be the result of an abrogated inflammatory environment (Chari et al., 2006; Clarner et al., 2011). This may in part be due to increases in the pro-inflammatory cytokine and tumor necrosis factor-alpha (TNF-α) in toxin-mediated demyelination that is produced by M1 macrophages (Voss et al., 2012; Sen et al., 2022). Furthermore, TNF-α has been shown to activate the plasma membrane phagocytic-triggering receptors expressed in myeloid cells 2 (TREM2) and expressed on microglia and macrophages, which in turn upregulates phagocytosis and suppresses interleukin 6 (IL6)-activated macrophages–necessary for the transition from acute to chronic inflammation. In addition, TREM2 can be activated by lipid ligands derived from myelin debris (Ferrara et al., 2022). In this way, TREM2-activated microglia are converted from an inflammatory phenotype to an anti-inflammatory one that potentiates neurorepair (Xu et al., 2021; Ferrara et al., 2022). The function of TREM2 in myelin debris clearance has been clinically highlighted through gene mutation and resulted in Nasu-Hakola disease–premature dementia and myelin loss (Xing et al., 2015).

Until recently, TREM2 was deemed “undruggable.” However, recent evidence determined TREM2 to be a positive endocrinologically regulated TH gene (Ferrara et al., 2022). Furthermore, activation of mTOR has shown to increase the viability of TREM2^+^ cells and permit long-term cell survival and growth in a mouse model of Alzheimer’s disease (Ulland et al., 2017).

This may be of great therapeutic benefit as there is evidence suggesting that genomic TH signaling is dampened in an inflammatory environment as observed in EAE-induced hypoxia. This has also been demonstrated to be a result of dysregulated TH transport (Davies et al., 2013). In addition to the association of HIF1-α with DIO3 in the SVZ-derived NPCs, abrogated TH signaling during neuroinflammation can initiate oligodendrogliopathy and delay myelination (Lee and Petratos, 2016). Hence, the activation of TREM2^+^ microglia may be regulated by TH and thyromimetics to promote neurorepair (Ferrara et al., 2022), and mTOR-regulated autophagy has been shown to polarize microglia to an M2 phenotype in sepsis-induced neuroinflammation (Zhuang et al., 2020).

However, competing evidence exists suggesting that the PI3K/Akt/mTOR signaling pathway is implicated in the upregulation of inducible nitric oxide synthase (iNOS) production under hypoxic conditions, which is associated with microglial activity consisting of an inflammatory phenotype (Weinstein et al., 2000; Lu et al., 2006). Nitric oxide (NO) is necessary for the dissolution of neuroinflammation and responsible for the induction of T-cell apoptosis (Zettl et al., 1997). To support this, apoptosis of T cells via microglial phagocytosis results in the downregulation of the inflammatory phenotype in microglia (Chan et al., 2006). Furthermore, complete inhibition of NOS-2 is associated with worsening clinical scores in EAE and suggests a beneficial threshold of NO production (Dalton and Wittmer, 2005). Thus, some interplay between mTOR regulation in both M1 and M2 phenotypes may exist to achieve homeostasis during myelin clearance and remyelination, and further research is required to elucidate this mechanism.

## Thyroid hormone-induced hypertrophy and mitochondrial metabolic regulation

The function of mTOR signaling in hypoxia is of continued significance because mTOR is implicated in cardiac hypertrophy in which hypoxia and oxidative stress predominate. TH can genomically signal via TH-sensitive promoter elements in nuclear-encoded mitochondrial genes during oxidative stress in myocardial cells. This can enhance mitochondrial biogenesis and alleviate the energetic demands of myocytes (Goldenthal et al., 2004; Marín-García, 2010). Iodothyronines, which are inclusive of TH analogs such as DITPA, induce similar pro-angiogenic and hypertrophic effects in infarcted heart tissue through non-genomic activation of cell surface receptor integrin αvβ3 that induces MAPK signaling (Bergh et al., 2005; Mousa et al., 2006) and it can be argued that this is a result of Akt/mTOR activation.

The effects seen in cardiac hypertrophy models showcase the ability of TH to genomically signal via TRα1 and TRβ1 and induce Akt/mTOR survival signaling, which confers cytoprotection and leads to increased synthesis of normal contractile proteins and hypertrophic gene upregulation (Ojamaa, 2010). A study conducted by Kuzman et al. (2007) supports the role of Akt/mTOR in the development of TH-induced cardiac hypertrophy as treatment with rapamycin in mice caused complete inhibition of hypertrophic development. Conversely, there is recent data suggesting that TH-deprivation imparts cardioprotective effects as TH addition led to maladaptive cardiac growth and mTOR upregulation in a mouse model (Kerp et al., 2021). However, this can be largely attributed to the altered adrenergic responsiveness of mouse hearts utilized in experiments that induced left-ventricular pressure overload, which is a pathological form of cardiac hypertrophy. It was determined that the hypertrophy was associated with G protein-coupled receptor (GPCR) activation as opposed to the physiological hypertrophy induced with TH treatment via Akt signaling that exhibits an increased myocardial mass in the absence of adverse effects (Dorn and Force, 2005; Ojamaa, 2010; Kerp et al., 2021) and thereby permits the cardioprotective nature of TH to remain largely unattested. Mitochondrial biogenesis pathways critical for axo-myelin integrity require effective Akt/mTOR signaling in OLs but specific pathway analysis dependent on TH and integrin activation remain unresolved (Fünfschilling et al., 2012).

However, the genomic signaling capacity of TH is not limited to the heart as a reduction in TRα-dependent transcription factors, such as peroxisome proliferator-activated receptor *γ* coactivator 1 alpha (PGC1α), mitochondrial transcription factor A (TFAM), and estrogen-related receptor *α* (ERRα), are all responsible for mitochondrial biogenesis as observed in the TRα mutation in skeletal muscle (Zhou et al., 2021). The upregulation of PGC-1α, which is the master transcriptional regulator of nuclear respiratory factors (NRFs), by TRα is also responsible for local DIO2 production in cells enhancing localized T3 production (Marín-García, 2010). Since the genomic activation of mTORC1 leads to an upregulation of PGC-1α associated with mitochondrial biogenesis, non-genomic activation of the Akt/mTOR pathway could provide beneficial genomic increases in local T3 production in OLs through an increase in mTORC1 signaling.

It should be noted that an increased rate of mitochondrial transcription and energy metabolism occurs in parallel with an increased production of reactive oxygen species (ROS), which are a by-product of the electron transport chain (ETC). Chronic exposure of mitochondrial DNA (mtDNA) to ROS, due to the proximity of mtDNA to oxidative machinery, can lead to the functional decline of mtDNA under conditions of oxidative stress (Ballinger et al., 2000; Tsutsui et al., 2009). This is particularly true in more severe cases of mitochondrial respiration, which involves the production of H_2_O_2_ (a major endogenous ROS) following electron leakage from the ETC (Chen et al., 2009). However, despite the potential of TH-induced mitochondrial biogenesis to induce such ROS-mediated damage, T3 protects from H_2_O_2_-induced oxidative stress via the PI3K-Akt signaling pathway through inhibition of mitochondrial ROS over-production both *in vivo* and *in vitro* (Zeng et al., 2019). Furthermore, T3-induced redox activation of c-Jun N-terminal kinase 2 alpha 2 (JNK2α2), which is a JNK member of the MAPK family, results in enhanced IGF-1 expression and activation of proliferative ERK1/2 signaling (Tan et al., 2019). Therefore, it is plausible that a non-canonical pathway activated by thyromimetic small molecules can induce OL proliferation. Actions of TH and thyromimetics, in the context of OL oxidative stress and mitochondrial biogenesis, may therefore drive remyelination by eliciting Akt/mTOR signaling and permit myelin biogenesis, neurorepair, and neuroprotection.

## Treatment for myelin diseases of the brain using thyroid hormone and thyromimetic analogs

Prior to neurotherapeutic advancements with thyromimetic analogs that include but are not limited to rT3, Sobetirome, tetraiodothyroacteic acid (TETRAC), triiodothyroacetic acid (TRIAC), and DITPA (Senese et al., 2019), individuals that presented with clinical symptoms related to TH deficiency or thyrotoxicosis were treated with TH to re-establish homeostasis of the HPT axis to reinstate proper functioning of tissues in the body (Wooliscroft et al., 2020).

In the context of neurodegenerative diseases where demyelinating events are prominent, provision of T3 and T4 were considered potential therapeutic interventions given their role in promoting myelination (Bernal, 2005). In cases related to local hypothyroidism and DIO2 dysregulation, peripheral immune cells secrete pro-inflammatory cytokines, such as interleukin-1β (IL-1β) and interferon-γ (IFN-γ), and cause an overall decrease of TH in the CNS that inhibits the differentiation of OLs (Merrill, 1991; Andrews et al., 1998; Boelen et al., 2004). Additionally, local administration of nanoparticles coated with TH were shown to reduce side-effects in the peripheral immune system and promote remyelination in the CNS (Mdzinarishvili et al., 2013).

In the EAE model of MS, the subcutaneous administration of T4 or T3 was demonstrated to ameliorate neurological deficits associated with the disease after onset (Fernandez et al., 2004; Calza et al., 2002; D'Intino et al., 2011). Similarly, in an EAE model using dark agouti rats co-treated with valproic acid, HDAC inhibitor, and T4 provision, there was an elevation in the expression of several myelin genes in O4^+^ (a surface antigen present in OLs) pre-OLs of the brain during the remission stage of EAE (Castelo-Branco et al., 2014b). Furthermore, a T4 treatment in rabbits (Vose et al., 2013) and rats (Hung et al., 2013) models associated with white matter damage predisposed to intraventricular hemorrhage and hypoxic ischemia-induced demyelination and inflammation, revealed an increase in myelination during early postnatal stages. These data were also supported by immunohistochemical analysis showing an increase in MBP expression after T4 and T3 administration and the promotion of increased myelination, which is a neuroprotective mechanism under neuroinflammatory conditions (Fernandez et al., 2004; Calza et al., 2002; D'Intino et al., 2011). The number of O4^+^/O1^+^ mature OLs increased via T4 administration in pre-term infants with intraventricular hemorrhage (IVH) compared to controls (Vose et al., 2013). Moreover, T3 administration through intraperitoneal (Harsan et al., 2008), subcutaneous (Franco et al., 2008) or intranasal (Silvestroff et al., 2012) delivery promoted remyelination in an MS-like animal model, i.e., the cuprizone-induced neurotoxicant model of demyelination and remyelination. Therefore, the preservation of OLs and remyelination may be a therapeutic option for repair of the CNS through TH administration (Sher et al., 2008; Kotter et al., 2011).

The development of TH analogs with thyromimetic effects in the periphery and CNS without off-target adverse events are essential to address therapeutic gaps in neuroprotection and repair related to TH signaling in white matter diseases.

The TH analog TRIAC is a natural metabolite found in the human body in lower concentrations than T3 (Menegay et al., 1989). Originally tested in male MCT8^−/−^ mice, TRIAC was administered to postnatal day 1–12 mice in doses between 50 and 400 ng/g/bodyweight. Results from this study demonstrated increased TH-dependent differentiation of neural precursors and cortical myelination (Horn et al., 2013a; Kersseboom et al., 2014). Studies assessing the safety of TRIAC administration were validated in TH-resistant patients by delivering 38.3 µg/kg/body weight to overcome thyrotoxicosis in male children with MCT8 deficiency (Groeneweg et al., 2017). However, studies by Groeneweg et al. (2017) and Anzai et al. (2012) also revealed a therapeutic caveat of TRIAC indicating that its half-life in humans was limited to just 6 h. As a corollary, TRIAC administration in mice deficient for MCT8 cannot upregulate thyroid hormone-responsive genes in the cerebral cortex and striatum, which suggests that despite the peripheral effects of this drug being clinically proven, it may be ineffective in promoting thyroid hormone-dependent signaling in the brain (Bárez-López et al., 2016).

Another TH analog, TETRAC, can also enter cells independent of MCT8. It was previously reported in studies of male MCT8^−/−^ mice that a daily injectable dose of TETRAC (400 ng/kg/body weight) in the first week during neonatal development successfully promoted neuronal development in the cerebral cortex, cerebellum, and striatum (Horn et al., 2013b). Unfortunately, from a translational standpoint, there is still limited pre-clinical evidence related to the mechanism of action, suggesting that TETRAC can be considered a safe and efficacious treatment option.

The synthetic TH analog DITPA has also been shown to enter cells in the brain independent of MCT8 and there is promising data indicating that DITPA can overcome MCT8 deficiencies (Lee et al., 2017). Additionally, it has been demonstrated that DITPA can successfully cross restrictive barriers such as the BBB and placenta, which make it an ideal candidate for antenatal treatment (Di Cosmo et al., 2009; Ferrara et al., 2014). In a noteworthy clinical trial of 4 children aged 8–25 months with MCT8 mutations, DITPA was administered in a dosing regimen starting at 1.8 mg/day and increasing to 30 mg/day (during 26–40 months). Results from this trial indicated that DITPA dosed at 1.2 mg/kg/day restored myelin to normal levels, improved weight gain, and sustained normal thyroid function. Additionally, there were no reports of hypothyroidism, thyrotoxicity, or other adverse events (Verge et al., 2012). These findings from pre-clinical *in vitro* and *in vivo* studies (Lee et al., 2017) support DITPA as an ideal candidate in treating white matter disorders associated by OL loss and/or dysmyelination, which occurs in the evolution of MS lesions.

TH analogs that do not require MCT8 have been suggested as a potential therapy to treat the X-linked psychomotor disorder Allan-Herndon-Dudley syndrome (AHDS). For example, DITPA can normalize peripheral hyperthyroidism and reduce hypermetabolism in AHDS patients. However, the exact mechanism by which DITPA acts is largely unknown. In the last few years, the off-label clinical trial (on compassionate grounds) reported by Verge et al. (2012) administered DITPA with an increasing dosage to a final maximum dose of 2–2.4 mg/kg per day over 15–29 months of treatment to 4 boys with AHDS (2 of which were monozygotic twin boys) and that ranged from 8.5 to 25 months of age. The analysis of these four boys over the 29-month treatment duration exhibited late myelination in the twin boys defined by repeat MRI imaging at 47 months-of-age. Outside beneficial normalization of hyperthyroid levels in the periphery, significant physiological changes were observed that included no weight loss and reduced sex hormone-binding globulin (SHBG) and heart rate. None of the children developed seizures or required assisted feeding through a gastric tube. However, a phase II clinical trial initiated by the Veterans Affairs Office of Research and Development (NCT00032643) in a randomized double blind interventional study for post-ischemic heart failure did report non-compliance in the DITPA treatment group with only 21 patients completing the study. This study reported adverse effects in this cohort of patients due to the clinical trial design that incorporated an increasing dose over the 24 week treatment period. This clinical trial highlighted the need for lower doses to ensure patient compliance and safety, but the therapeutic dose required to demonstrate efficacy for CNS pathology requires further elucidation.

## Conclusion

MS is a chronic, neurodegenerative disease that is characterized by autoimmune inflammatory lesions located within the CNS. However, the etiology of MS is not well defined, and current therapeutic approaches that are effective in MS treatment can only address the relapse rate in individuals living with MS. However, recent research has determined that drugs that target and elicit TH-dependent mechanisms within OLs may hold clinical promise during acute inflammatory demyelination by independently activating these mature cells along with their lineage precursor population. Due to their ability to bypass the monocarboxylate transporter 8, TH analogs such as TRIAC, TETRAC, and DITPA may be effective at promoting mature PLP^+^ mature oligodendrocyte neuroprotection and may potentiate the migration of OPCs that display acute downregulation of MCT8 as a consequence of neuroinflammatory disease. Moreover, oligodendroglial cells that are targeted through TH analogs may occur through the activation of the downstream P-Akt/P-mTOR pathway that promotes oligodendroglial cell viability and maturation. Neurological recovery following TH analog administration that is a direct result of the preservation of oligodendrocytes and/or remyelination needs to be clearly documented in the context of a neuroinflammatory challenge. Importantly, we describe an alternate TH-dependent mechanism of action in targeted oligodendroglial cells by outlining the binding and activation through integrin receptors to signal through the non-genomic PI3K/Akt/mTOR pathway facilitating cell viability and maturation. Further studies defining the function of MCT8 during the mobilization and differentiation of OPCs during neuroinflammation are required and may identify how OPCs can be metabolically salvaged and stimulate differentiation during MS and other diseases with profound myelin pathology. These approaches modify the TH-dependent non-genomic signaling pathways and may permit endogenous repair of demyelinated lesions, and ultimately, potentiate neurological recovery.

## Glossary

AHDS: Allan-Herndon-Dudley syndrome
APC: Antigen-presenting cell
Akt: Ak strain transforming
BBB: Blood-brain barrier
CNS: Central nervous system
CNP: 2′, 3′-Cyclic-nucleotide-3′-phosphodiesterase
CoA: Co-activator protein
CoR: Co-repressor protein
cc: Corpus callosum
DMTs: Disease-modifying therapies
DITPA: 3, 5-diiodothyropropionic acid
DIO: Deiodinase
EAE: Experimental autoimmune encephalitis
EDSS: Expanded disability status scale
ECM: Extracellular matrix
ERK: Extracellular signal-regulated kinase
ETC: Electron transport chain
ERR: Estrogen-related receptor
FGF: Fibroblast growth factor
GAG: Glycosaminoglycans
GFAP: Glial fibrillary acidic protein
GPCR: G protein-coupled receptor
GTPase: Guanosine triphosphate hydrolase
HDAC: Histone deacetylase
HI: Hypoxic-ischemic
HIF-1α: hypoxia-inducible factor-1 alpha
HLA-DR15: HLA-DRB1*15:01-DQB1*602
IGF: Insulin growth factor
IFN-β: Interferon-β
iNOS: Inducible nitric oxide synthase
IL-1β: Interleukin-1β
IRS: Insulin receptor substrate
JNK2α2: c-Jun N-terminal kinase 2 alpha 2
LAT: L-type amino acid transporter
LN: Laminin
MAPK: Mitogen-activated protein kinase
MBP: Myelin basic proteins
MCT8: Monocarboxylate transporter 8
MCT10: Monocarboxylate transporter 10
MOG: Myelin oligodendrocyte protein
MRI: Magnetic resonance imaging
MS: Multiple sclerosis
mtDNA: Mitochondrial DNA
mTOR: Mammalian target of rapamycin
mTORC1: Mammalian target of rapamycin complex 1
mTORC2: Mammalian target of rapamycin complex 2
NPCs: Neural precursor cells
NRF: Nuclear respiratory factor
OAT: Organic anion transporter
OL: Oligodendrocyte
OPC: Oligodendrocyte progenitor cell
PDGF: Platelet-derived growth factor
PDK1: Phosphoinositide-dependent kinase-1
PGC1: Proliferator-activated *γ* receptor coactivator 1
PI3K: Phosphoinositide 3-kinase
PLP: Proteolipid protein
PPMS: Primary progressive multiple sclerosis
PRMS: Progressive-relapsing multiple sclerosis
*pt*: Pertussin toxin
PTEN: Phosphatase and tensin homolog
PTPα: Protein tyrosine phosphatase alpha
RGD: Arginylglycylaspartic acid
Rhes: Ras family GTPase
ROS: Reactive oxygen species
RRMS: Relapsing-remitting multiple sclerosis
RTK: Receptor tyrosine kinase
RXR: Retinoid-X-receptors
SHBG: Sex hormone-binding globulin
SPMS: Secondary progressive multiple sclerosis
SVZ: Sub-ventricular zone
T3: Triiodothyronine
T4: Thyroxine
TETRAC: Tetraiodothyroacteic acid
TFAM: Mitochondrial transcription factor
TREM2: Triggering receptors expressed in myeloid cells 2
TRIAC: Triiodothyroacetic acid
TSC: Tuberous sclerosis complex
TGA: Therapeutic Goods Administration
THT: Thyroid hormone transporter
TH: Thyroid hormone
TNF: Tumor necrosis factor
TR: Thyroid hormone receptor
TRE: Thyroid hormone response elements
TRH: Thyrotropin-releasing hormone
